# Selective Serotonin Reuptake Inhibitor Pharmaco-Omics: Mechanisms and Prediction

**DOI:** 10.3389/fphar.2020.614048

**Published:** 2021-01-11

**Authors:** Thanh Thanh L. Nguyen, Duan Liu, Ming-Fen Ho, Arjun P. Athreya, Richard Weinshilboum

**Affiliations:** ^1^ Division of Clinical Pharmacology, Department of Molecular Pharmacology and Experimental Therapeutics, Mayo Clinic, Rochester, MN, United States; ^2^ Graduate School of Biomedical Sciences, Mayo Clinic, Rochester, MN, United States

**Keywords:** selective serotonergic reuptake inhibitors, major depressive disorder, serotonin, kynurenine, pharmacogenomics, pharmaco-omics, machine learning, predictive algorithm

## Abstract

Selective serotonin reuptake inhibitors (SSRIs) are a standard of care for the pharmacotherapy of patients suffering from Major Depressive Disorder (MDD). However, only one-half to two-thirds of MDD patients respond to SSRI therapy. Recently, a “multiple omics” research strategy was applied to identify genetic differences between patients who did and did not respond to SSRI therapy. As a first step, plasma metabolites were assayed using samples from the 803 patients in the PGRN-AMPS SSRI MDD trial. The metabolomics data were then used to “inform” genomics by performing a genome-wide association study (GWAS) for plasma concentrations of the metabolite most highly associated with clinical response, serotonin (5-HT). Two genome-wide or near genome-wide significant single nucleotide polymorphism (SNP) signals were identified, one that mapped near the *TSPAN5* gene and another across the *ERICH3* gene, both genes that are highly expressed in the brain. Knocking down TSPAN5 and ERICH3 resulted in decreased 5-HT concentrations in neuroblastoma cell culture media and decreased expression of enzymes involved in 5-HT biosynthesis and metabolism. Functional genomic studies demonstrated that ERICH3 was involved in clathrin-mediated vesicle formation and *TSPAN5* was an ethanol-responsive gene that may be a marker for response to acamprosate pharmacotherapy of alcohol use disorder (AUD), a neuropsychiatric disorder highly co-morbid with MDD. In parallel studies, kynurenine was the plasma metabolite most highly associated with MDD symptom severity and application of a metabolomics-informed pharmacogenomics approach identified *DEFB1* and *AHR* as genes associated with variation in plasma kynurenine levels. Both genes also contributed to kynurenine-related inflammatory pathways. Finally, a multiply replicated predictive algorithm for SSRI clinical response with a balanced predictive accuracy of 76% (compared with 56% for clinical data alone) was developed by including the SNPs in *TSPAN5*, *ERICH3*, *DEFB1* and *AHR*. In summary, application of a multiple omics research strategy that used metabolomics to inform genomics, followed by functional genomic studies, identified novel genes that influenced monoamine biology and made it possible to develop a predictive algorithm for SSRI clinical outcomes in MDD. A similar pharmaco-omic research strategy might be broadly applicable for the study of other neuropsychiatric diseases and their drug therapy.

## Introduction: Pharmacogenomics to Pharmaco-Omics

**FIGURE 1:**
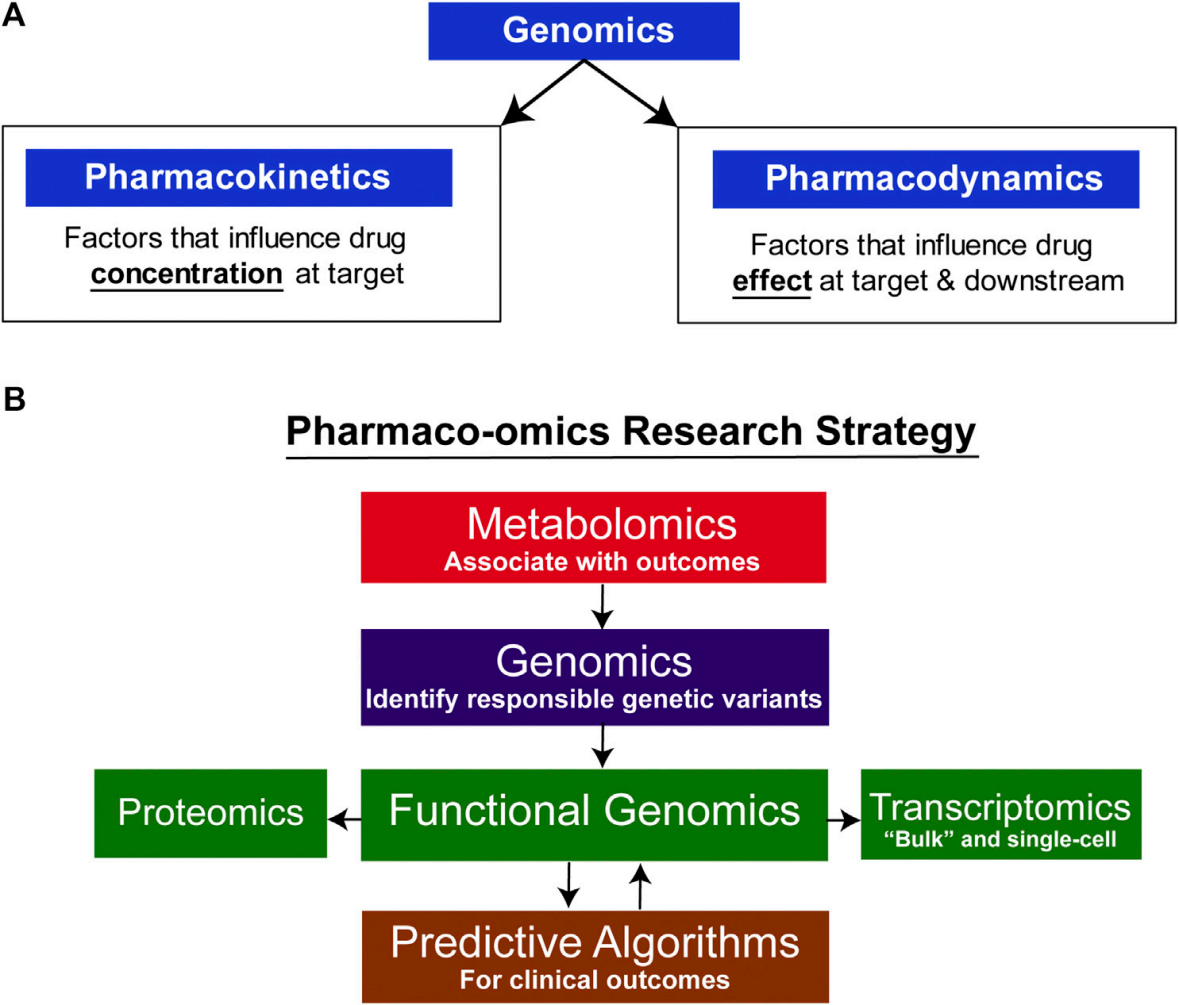
Pharmaco-omic concepts. **(A)** Genomic factors can influence both pharmacokinetic and pharmacodynamic aspects of drug therapy. **(B)** Pharmaco-omics Research Strategy. Variation in drug response in this series of studies was initially associated with variation in metabolite concentrations, and GWAS were then conducted using those concentrations as phenotypes. A series of functional genomic studies was then performed. Finally, machine learning algorithms were developed using both clinical information and SNPs for the top hits in the GWA studies. The algorithms also informed further functional genomic studies by identifying the most significant contributors to prediction accuracy.

Pharmacogenomics (PGx), the study of the role of inheritance in individual variation in drug response, has evolved from early “pharmacogenetic” studies of candidate genes, often genes encoding drug metabolizing enzymes, to become “pharmacogenomics” after it became possible to scan across the genome in an unbiased fashion to identify genes associated with variation in drug response (Wang et al., 2011; Weinshilboum and Wang, 2017). Variation in drug response can result from variation in either “pharmacokinetics,” factors that influence the concentration of drug that reaches its target, or “pharmacodynamics,” factors involving the drug target itself or processes downstream of the target (see Figure 1A) (Wang et al., 2011; Weinshilboum and Wang, 2017). Early examples of pharmacogenetics often involved genes that encoded drug metabolizing enzymes or drug transporters, genes that were obvious candidates for study. Psychiatry and psychopharmacology participated actively in the early development of pharmacogenetics with reports decades ago of genetic variation in human genes encoding proteins of importance for psychiatry such as the catecholamine metabolizing enzyme catechol O-methyltranferase (*COMT*) (Weinshilboum and Raymond, 1977; Scanlon et al., 1979; Ho and Weinshilboum, 2019) and the catecholamine biosynthetic enzyme dopamine beta-hydroxylase (*DBH*) (Weinshilboum et al., 1975; Dunnette and Weinshilboum, 1977). Psychopharmacology also led the way in reports of genetic variation in genes encoding important drug metabolizing enzymes such as *CYP2D6*—an enzyme that plays a major role in the biotransformation of many drugs including SSRIs (Johansson et al., 1993). Recently, rapid advances in “-omic” technologies, e.g., metabolomics, transcriptomics and proteomics, coupled with a computational revolution that has made it possible to integrate and analyze large datasets, have enabled pharmacogenomics to expand beyond the genome to become “pharmaco-omics”—as will be illustrated by the subsequent description of SSRI pharmaco-omics (see Figure 1B). Specifically, this brief review will describe the application of a multiple omics research strategy in an attempt to increase our understanding of and our ability to predict variation in clinical response for an extremely important class of drugs, the selective serotonin reuptake inhibitors (SSRIs).

## SSRI Pharmacometabolomics-Informed Pharmacogenomics

Major depressive disorder (MDD) is a common and potentially devastating psychiatric disorder with a lifetime prevalence of approximately 13% worldwide (Alonso et al., 2004; Bromet et al., 2011). Even though the pathophysiology of MDD is not fully understood, a relative deficiency of serotonin (5-HT) and other central nervous system (CNS) monoamine neurotransmitters clearly plays an important role (Morrissette and Stahl, 2014). SSRIs, drugs that enhance serotonergic neurotransmission, have become first-line pharmacotherapy for the treatment of MDD (Clevenger et al., 2018; Kato et al., 2018). However, only approximately one half to two-thirds of MDD patients respond to SSRI therapy, and that response may require weeks or months to develop (Trivedi et al., 2006). As a result, greater understanding of mechanism(s) underlying individual variation in SSRI clinical response remains a major goal of antidepressant research. It had been hoped that genome-wide association studies (GWAS) would provide novel insight into both underlying molecular causes of MDD and variation in MDD drug response. Unfortunately, the use of GWAS to study variation in SSRI response has met with only limited replicated success (Garriock et al., 2010; Uher et al., 2010; Ji et al., 2013; Biernacka et al., 2016). In part, that may be due to underlying biological heterogeneity of MDD as well as a lack of validated biomarkers for this disease (Krishnan and Nestler, 2008). The development of very large clinical datasets joined to genome-wide genomic data, for example, the United Kingdom Biobank (Bycroft et al., 2018), has provided novel insight into molecular risk for many diseases, but it has been less successful when applied to drug response because the extraction of accurate information with regard to drug use and response from medical records and electronic health records (EHRs) has been challenging. As a result, it is important that we develop novel research strategies to take advantage of technical advances in molecular assays and new methods of data analysis such as machine learning and artificial intelligence—as described in subsequent paragraphs.

In an attempt to address the challenge presented by individual variation in SSRI clinical response, the Mayo Clinic Pharmacogenomics Research Network-Antidepressant Medication Pharmacogenomics Study (PGRN-AMPS) applied a metabolomics-informed genomics research strategy to study samples from that 803 patient MDD SSRI trial (Mrazek et al., 2014). This approach began by associating plasma metabolite concentrations with symptom severity before and after drug treatment, followed by GWAS for concentrations of the metabolites that were significantly associated with SSRI treatment outcomes to identify genetic polymorphisms responsible for variation in metabolite concentrations (Gupta et al., 2016; Neavin et al., 2016; Liu et al., 2018) (see Figure 1B). The hypothesis underlying this metabolomics-informed pharmacogenomic approach was that genes identified in this fashion might also be associated with MDD pathophysiology and/or variation in SSRI response. Metabolite concentrations are quantitative biological traits and, as a result, they differ from the rating scales used to help diagnose and evaluate treatment response in psychiatry. However, it is important to note that metabolite concentrations in blood can fluctuate in response to environmental variables beyond genomics, a limitation that always needs to be recognized and acknowledged. Additionally, there is no assurance that the regulation of metabolite concentrations in the periphery is similar to that in the CNS. Therefore, as described subsequently, a series of functional studies using cell lines that originated from or were differentiated to resemble CNS cells were conducted to study the function of genes identified as a result of their association with metabolite concentrations, making it possible to draw parallels between regulatory mechanisms in the CNS and the periphery.

**FIGURE 2:**
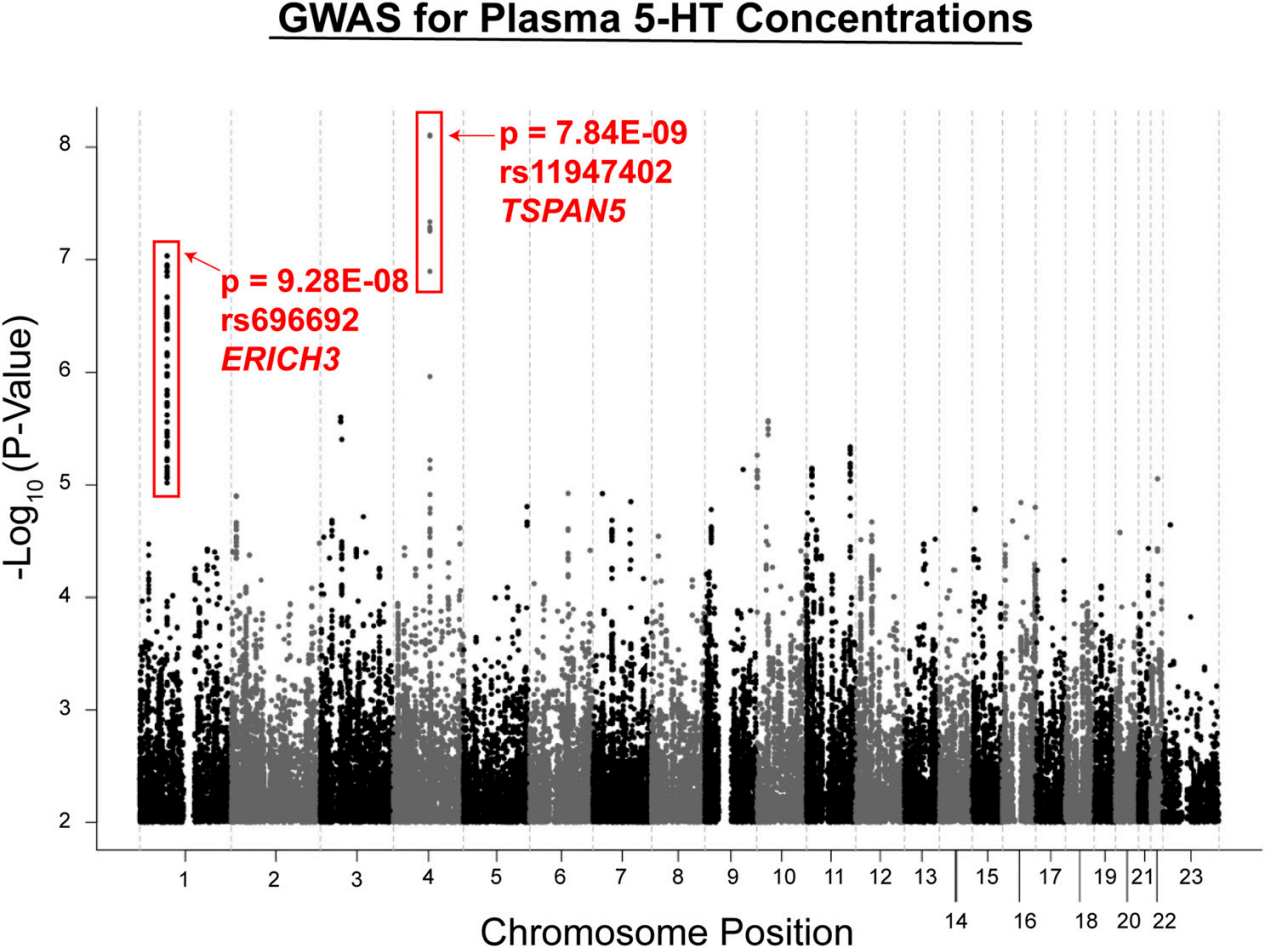
GWAS for plasma serotonin concentrations in the PGRN-AMPS trial. The Manhattan plot shows results for a GWAS for plasma serotonin concentrations at baseline in the PGRN-AMPS MDD patients who were studied. The Y axis represents−log 10 of *p*-values, and the X axis represents chromosomal position. Each dot represents a SNP. The figure was adapted from Gupta et al., 2016.

Metabolite concentrations were assayed using plasma from MDD patients at baseline and after 4 and 8 weeks of therapy with citalopram or escitalopram, two structurally related SSRIs, using a “targeted” metabolomics platform with high sensitivity for monoamine neurotransmitters or their metabolites (Gupta et al., 2016). A targeted platform was used because broader platforms were not always quantitative, and we used a liquid chromatography electrochemical array (LCECA) to detect the metabolites because of its greatly superior sensitivity for monoamine transmitters and their metabolites (Mark et al., 1984). However, the limitation of this approach—which should be kept in mind—is that it will fail to detect compounds that do not display an electrochemical signal. The plasma metabolite that was most highly associated with SSRI response, either Remission (HAMD ≤ 7 or QIDS-16C ≤ 5) or Response (≥ 50% decrease in either HAMD or QIDS-16C without achieving Remission) was serotonin (5-hydroxytryptamine, 5-HT). GWAS for plasma 5-HT in the 290 patients studied identified two genome-wide significant or near genome-wide significant SNP signals (see the Manhattan plot in Figure 2). Specifically, one SNP signal mapped 5′ of the Tetraspanin 5 (*TSPAN5*) gene on chromosome 4 (*p* = 7.84*E* − 09), and the other mapped across the glutamate-rich 3 (*ERICH3*) gene on chromosome 1 (*p* = 9.28*E* − 08) (Gupta et al., 2016). Both of these genes were highly expressed in the brain. The *TSPAN5* SNPs were expression quantitative trait loci (eQTLs) for *TSPAN5*, that is, they were associated with mRNA expression of the gene in a SNP genotype-dependent fashion in multiple tissues including the brain according to the Genotype-Tissue Expression database (GTEx) (GTExConsortium et al., 2017). The *ERICH3* SNPs, on the other hand, were associated with decreased *ERICH3* expression at the protein level, probably as a result of accelerated degradation of the variant allozymes (Gupta et al., 2016). The “top” SNP for *ERICH3* was also associated with SSRI response for MDD patients enrolled in other large clinical trials (Liu et al., 2020) including the Sequenced Treatment Alternatives to Relieve Depression (STAR*D) (Trivedi et al., 2006), the International SSRI Pharmacogenomics Consortium (ISPC) (Biernacka et al., 2016), and the Predicting Response to Depression Treatment Test (PReDICT) (Dunlop et al., 2012; Dunlop et al., 2017) trials. Recently, other SNPs that mapped to *ERICH3* were found to be associated with MDD risk in the United Kingdom Biobank repository (McInnes et al., 2019).

**FIGURE 3:**
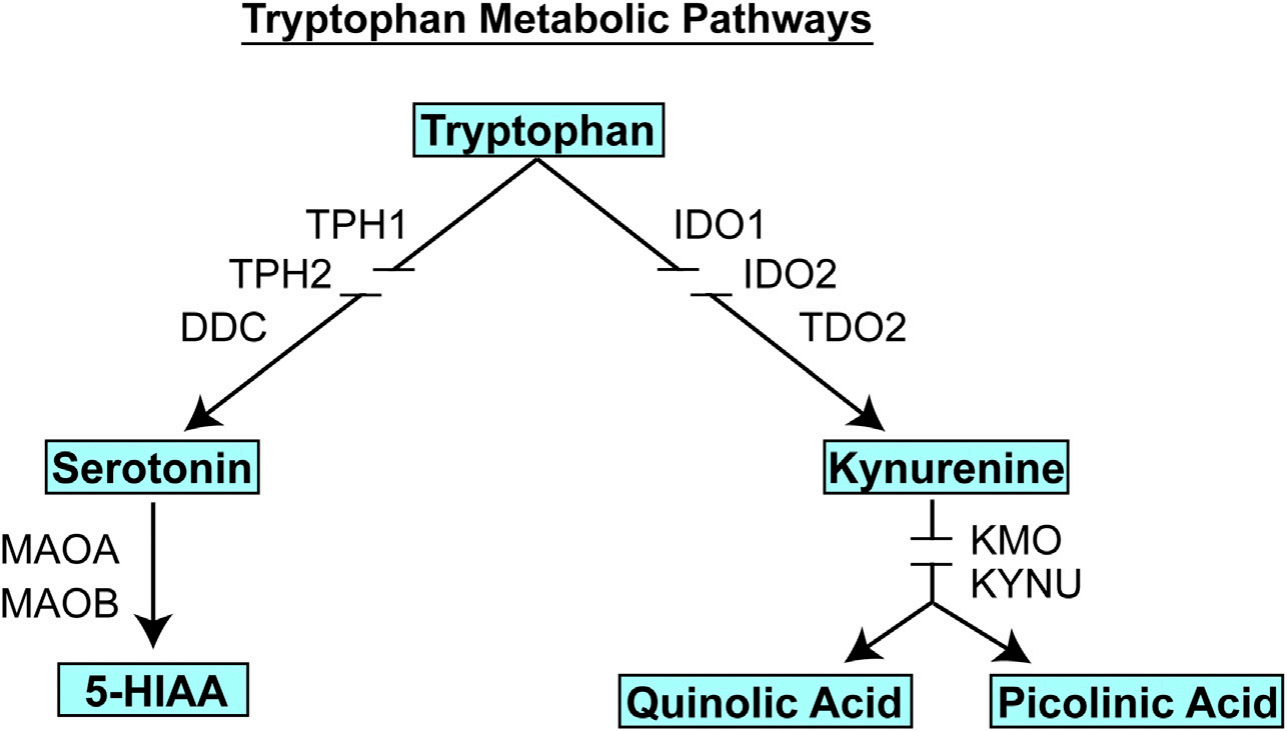
Tryptophan metabolic pathways. Tryptophan is metabolized to form, among other compounds, either serotonin (5-HT) or kynurenine. Gene name abbreviations: TPH1, Tryptophan Hydroxylase 1; TPH2, Tryptophan Hydroxylase 2; DDC, Dopa Decarboxylase; MAOA, monoamine oxidase A, MAOB, monoamine oxidase B; IDO1, indoleamine 2,3-dioxygenase 1; IDO2, indoleamine 2,3-dioxygenase 2; TDO2, tryptophan 2,3-dioxygenase, KMO, kynurenine 3-monooxygenase; KYNU, kynureninase.

Subsequent studies in which an identical research strategy was applied to severity of MDD symptoms among the patients enrolled in the PGRN-AMPS SSRI trial rather than variation in SSRI response found that kynurenine, an endogenous compound that, like 5-HT, is a metabolite of the amino acid tryptophan (see Figure 3), was the metabolite most highly associated with disease severity as determined by either HAMD or QIDS-16C scores (Liu et al., 2018). GWAS for plasma kynurenine concentrations identified two SNP signals, one of which mapped across the beta-defensin 1 (*DEFB1*) gene (*p* = 8.18*E* − 07), while the other mapped across the aryl hydrocarbon receptor (*AHR*) gene (*p* = 6.22*E* = 06) (Liu et al., 2018). The SNPs in both cases were eQTLs for DEFB1 and AHR, respectively. While these SNPs were not genome-wide significant, functional studies, as described subsequently, demonstrated that both DEFB1 and AHR played important roles in mediating inflammatory pathways that are involved in depression (Herbert and Cohen, 1993; Bufalino et al., 2013). Furthermore, when we describe the development of a machine learning-based, multiply replicated predictive algorithm for SSRI response in MDD, it was found that SNPs from all four of the signals identified during these two GWAS, those for TSPAN5, ERICH3, DEFB1 and AHR, all contributed to the predictive accuracy of the algorithm (Athreya et al., 2018; Athreya et al., 2019b). Even though the sample size for the initial GWA studies was small, the use of metabolomics to inform genomics resulted in genome-wide or near genome-wide significant signals, several of which were replicated in other trials, as mentioned above, and the incorporation of SNPs from all of these signals contributed to the predictive accuracy of the algorithm described subsequently.

## Functional Genomics

**FIGURE 4:**
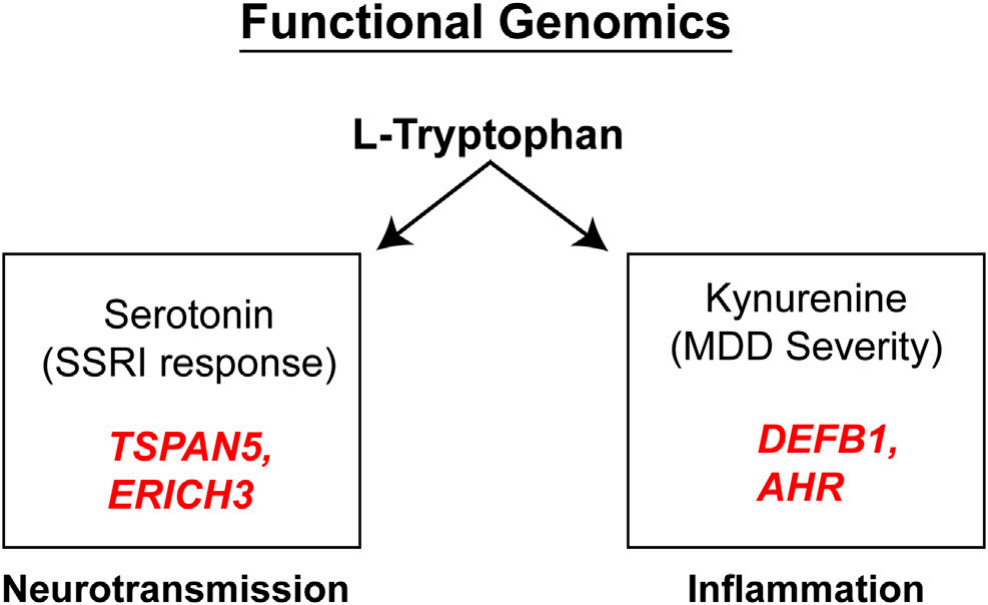
Functional genomics. Functional genomic studies of Tetraspanin 5 (*TSPAN5*) and Glutamate Rich 3 (*ERICH3*), genes identified during the 5-HT GWAS, highlighted their role in monoamine transmission while the Beta-Defensin 1 (*DEFB1*) and Aryl Hydrocarbon Receptor (*AHR*) genes identified during the kynurenine GWAS, were both related to inflammation.

It should be emphasized that the genes identified in the course of the GWA studies performed using plasma metabolites as phenotypes did not encode enzymes involved in either the biosynthesis or metabolism of those metabolites. We mention this fact because it indicates that the variation in plasma concentrations of both 5-HT and kynurenine, variation that was associated with SSRI response and severity of MDD symptoms (see Figure 4), respectively, appeared to be associated with the effects of proteins, TSPAN5, ERICH3, DEFB1 and AHR, which had not previously figured prominently--or at all--in our thinking with regard to MDD pathophysiologic mechanism(s). Therefore, after their identification, it was necessary that a series of functional genomic studies be performed in an attempt to make it possible to better understand biological mechanisms underlying individual variation in concentrations of these plasma metabolites, metabolites that were themselves associated with SSRI clinical response and—potentially—mechanisms related to MDD pathophysiology. Prior to moving to the functional genomic results, it should be emphasized once again that both 5-HT and kynurenine are downstream metabolites of tryptophan, as depicted graphically in Figure 3.

### TSPAN5: 5-HT, Kynurenine and MDD-AUD Cross-Talk

Knock-down (KD) and overexpression (OE) of TSPAN5 in neuroblastoma cells significantly altered the expression of serotonin biosynthetic and metabolizing enzymes including TPH1, TPH2, DDC and MAOA, resulting in altered 5-HT concentrations in the cell culture media (Gupta et al., 2016). Specifically, KD lowered the expression of these enzymes, while OE resulted in elevated expression. In 2020, Ho et al., replicated these observations using forebrain neurons and astrocytes derived from human induced-pluripotent stem cells (iPSCs) (Ho et al., 2020), better models for human CNS cells than were the neuroblastoma cell lines studied in the original 2016 experiments. KD of TSPAN5 also influenced kynurenine concentrations as well as a series of immune response signaling pathways based on RNA-sequencing results (Ho et al., 2020). Of particular interest was the fact that *TSPAN5* was found to be an alcohol-responsive gene that may play a role in alcohol use disorder (AUD) pharmacotherapy with the anti-craving drug acamprosate (Ho et al., 2020)—one of only three drugs approved by the United States Food and Drug Administration for the treatment of AUD. Specifically, both ethanol and acamprosate, at physiologically appropriate concentrations, down-regulated the expression of TSPAN5 as well as that of genes encoding enzymes in the serotonin and kynurenine metabolic pathways from tryptophan (Ho et al., 2020). Of particular importance, a cluster of *TSPAN5* SNPs was found to be associated with length of abstinence during 3 months of acamprosate treatment for AUD patients enrolled in the Mayo Clinic Center for the Individualized Treatment of Alcoholism clinical trial (Ho et al., 2020). Taken together, these results raise the possibility that TSPAN5 SNPs might be biomarkers for acamprosate treatment response, and that the gene itself might play a role in cross-talk between MDD and AUD, two psychiatric disorders with high co-morbidity and, perhaps, partially shared pathophysiology (Kendler et al., 1993; Walters et al., 2018; Gelernter et al., 2019).

### ERICH3: Vesicular Function and Neurotransmitters

At the time of the discovery of the association of SNPs across *ERICH3* with plasma 5-HT concentrations in MDD patients treated with SSRIs, *ERICH3* was an uncharacterized gene that was most highly expressed in the human brain according to the GTEx database (GTExConsortium et al., 2017). Transcriptomic analysis of human brain tissue single-cell RNA-seq showed that *ERICH3* is expressed predominantly in neurons rather than other CNS cell types (Tasic et al., 2018). The *ERICH3* gene expresses multiple splice variants (Liu et al., 2020). Co-immunoprecipitation of ERICH3 protein, followed by mass spectrometric identification of interacting proteins showed that ERICH3 interacted with a series of neurotransmitter vesicular-associated proteins including CLTC, AP2A2, and PIK3C2A (Liu et al., 2020). It also interacted with ALK and CUX1 (Liu et al., 2020), proteins encoded by genes with genetic polymorphisms that have been reported to be associated with antidepressant treatment response (Ji et al., 2013; Sasayama et al., 2013), although with unknown molecular mechanism(s). KD of *ERICH3* resulted in decreased 5-HT concentrations in both cell culture media and cell lysates. These observations was replicated in an *ERICH3* knock-out experiment using CRISPR/Cas9 (Liu et al., 2020). Taken together, this series of observations raises the possibility that ERICH3 might influence 5-HT concentrations as a result of alterations in neurotransmitter vesicular function (Liu et al., 2020). Of note, the functional implications of ERICH3 might extend beyond serotonin to include other neurotransmitters since the co-localization of ERICH3 and dopamine in dopaminergic neurons was also observed (Liu et al., 2020), and independent single-cell RNA-seq data for human cerebral cortex showed that ERICH3 was also expressed in human glutamatergic and GABAergic neurons (Tasic et al., 2018). Finally, it is important to note that *ERICH3* was identified by a GWAS for a phenotype based on variation in 5-HT concentrations in patient blood samples. *ERICH3* is not only highly expressed in the brain, but it is also expressed in platelets where 99% of the 5-HT in the blood is stored in granules (Crane et al., 2015; Gehin et al., 2018). Mechanisms by which ERICH3 influences 5-HT concentrations in plasma—probably through effects on platelet SLC6A4, the serotonin transporter that is the target for SSRI drugs, might reflect similar mechanism in the CNS, as explained in a recent publication describing *ERICH3* functional genomics (Liu et al., 2020). This series of observations suggests that insights gained from experimental samples obtained in the periphery, in blood plasma, can potentially provide insight into physiological processes in the brain, in this case, the function of a novel gene with potentially important functions in SSRI response as well as in MDD pathophysiology.

### Studies of Serotonergic Circuitry in Patient-Derived iPSC Generated Serotonergic Neurons

The development of pluripotent stem cells and the differentiation of iPSCs into neuronal cell types have offered novel tools for the study of molecular mechanisms underlying CNS disease including psychiatric disorders. As mentioned above, in the functional genomic studies of the 5-HT and kynurenine metabolomics GWAS signals, iPSC-derived neurons of various types and iSPC-derived astrocytes were used to validate and expand initial molecular findings obtained from cell line models such as neuroblastoma cells. Those experiments utilized iPSC-derived neural cells to directly investigate gene function on an isogenic background, that is, by knocking-out the gene of interest using CRISPR/Cas9 and then assessing downstream phenotypes in the same cell line. A complementary approach would involve the generation of iPSCs from MDD patients who did and did not respond to SSRI therapy, followed by their differentiation into appropriate neuronal cell lines and investigation of molecular mechanisms that might contribute to differences in drug response. Because SSRIs target serotonin reuptake, a potentially useful *in vitro* model would be iPSC-derived serotonergic neurons, a technology that was developed only recently (Lu et al., 2016; Vadodaria et al., 2016; Xu et al., 2016). Specifically, using skin biopsies from MDD patients enrolled in the PGRN-AMPS trial--three female SSRI responders and three female non-responders, iPSCs were generated and differentiated into functional serotonergic neurons (Vadodaria et al., 2019a; Vadodaria et al., 2019b). Altered neurite growth, morphology (Vadodaria et al., 2019b), and hyperactivity downstream of upregulated excitatory serotonergic receptors (Vadodaria et al., 2019a) were observed in non-responding patient-derived serotonergic neurons. RNA-sequencing showed that non-responding patient-derived neurons had decreased expression of the protocadherin alpha genes that are involved in the regulation of neurite length and morphology (Vadodaria et al., 2019b). Those results suggested that altered serotonergic circuitry in non-responding patients might represent one factor involved in resistance to SSRI therapy. It is important to acknowledge the inherent challenges involved in studying non-isogenic iPSCs, since these cells are known to display considerable phenotypic variation among colonies as well as among cell lines (Cahan and Daley, 2013). To address that variation would require a large number of cell lines, an effort which, when coupled with the extensive resources and time required for the differentiation of pure serotonergic neurons, would be challenging.

### DEFB1: The Gut-Brain Axis and Inflammation

Just as 5-HT was the metabolite among those assayed that showed the highest association with SSRI response in the group of PGRN-AMPS SSRI trial subjects for whom metabolomic assays were performed, the metabolite that was most highly associated with severity of MDD symptoms as determined by HAMD or QIDS-C16 scores was kynurenine (Liu et al., 2018). When a GWAS was performed for baseline plasma kynurenine in these MDD patients, two SNP signals that mapped to the *DEFB1* and *AHR* genes were identified (Liu et al., 2018). Beta-defensing 1 encoded by the *DEFB1* gene is an antimicrobial gut mucosal protein associated with innate immunity and bacterial infection-induced inflammation—both of which have been associated with depression (Herbert and Cohen, 1993; Bufalino et al., 2013). Furthermore, AHR is known to regulate kynurenine biosynthesis (Neavin et al., 2018) and, as mentioned previously, both 5-HT and kynurenine are downstream metabolites of the amino acid tryptophan (see Figure 3). Therefore, even though the *p* values for these two gene signals that were identified during the kynurenine GWAS were not genome-wide significant, both signals were pursued functionally. Additionally, as pointed out subsequently when the MDD SSRI treatment outcomes machine learning-based predictive algorithm is described, it was fortunate that these genes were pursued since SNPs in both genes contributed significantly to the accuracy of that predictive algorithm (Athreya et al., 2019b).

Kynurenine can cross the blood-brain barrier and approximately 60% of the kynurenine in the brain is synthesized in the liver (Schwarcz et al., 2012). The enzyme primarily responsible for kynurenine synthesis in the liver is TDO2, while the synthesis of kynurenine in immune cells is primarily catalyzed by IDO1 and IDO2 (see Figure 3). Using THP-1 monocytic cells as a model system, functional genomic studies of DEFB1 showed that the inflammatory mediator liposaccharide (LPS) could induce the expression of IDO1 in these cells, but that the addition of recombinant DEFB1 strongly inhibited that induction (Liu et al., 2018). In parallel, during LPS induction of IDO1, culture media concentrations of tryptophan decreased while kynurenine concentrations increased and, in both situations, those effects were significantly “blunted” by the addition of recombinant DEFB1. These observations were compatible with the conclusion that DEFB1 is capable of influencing the biosynthesis of kynurenine and plasma kynurenine concentrations were the phenotype for the GWAS which initially identified the *DEFB1* gene (Liu et al., 2018). Finally, one of the *DEFB1* SNPs, rs2702877, was significantly associated with severity of MDD symptoms using data from all 803 MDD patients enrolled in the PGRN-AMPS SSRI clinical trial, both on the basis of HAMD-17 scores (*p* = 1.74E − 04) and QIDS-C16 scores (*p* = 1.25E − 05). These observations with regard to DEFB1 fit well with the rapidly evolving concept of a “microbiota-gut-brain” axis (Mayer, 2011; Cryan and Dinan, 2012). As described subsequently, very similar results were found with regard to the influence of genetic differences in AHR expression on kynurenine biosynthesis and their relationship to TDO2 expression in the liver based on the results of studies performed with HepaRG cells as a model system for the synthesis of hepatic kynurenine.

### AHR: Regulation of Kynurenine Biosynthesis and Metabolism

AHR is a ligand activated transcription factor (Neavin et al., 2018) and—as mentioned previously—the majority of kynurenine in plasma is synthesized in the liver, with approximately 60% of the kynurenine in the brain originating in the liver (Schwarcz et al., 2012). HepaRG cells are liver progenitor cells that can be differentiated into hepatocyte-like cells. When siRNA was used to KD AHR expression in these cells, the expression of TDO2, the major hepatic enzyme that catalyzes kynurenine biosynthesis, was greatly increased as was the expression of KMO and KYNU, enzymes in the pathway downstream from kynurenine, indicating that AHR appeared to repress the expression of all three of these genes (see Figure 3) (Liu et al., 2018). Conversely, when AHR was activated by exposure to an AHR ligand, 3-methylcholanthrene (3-MC), the expression of all three of these genes decreased significantly (Liu et al., 2018). In both situations, the changes in expression were observed at both the mRNA and protein levels. However, cell culture media kynurenine concentrations decreased after AHR KD, probably because of increased downstream metabolism catalyzed by KMO and KYNU. These observations provide insight into the functional consequences of the eQTL SNPs in the *AHR* gene, SNPs that were associated with decreased AHR expression. The subsequent section of this brief review merges the results of the GWAS and functional genomic studies and describes how the utilization of machine learning integrating those data made it possible to develop a predictive algorithm for SSRI response in MDD patients.

## Predicting SSRI Response

In psychiatry, measurements of disease severity prior to and clinical outcomes after therapy are based on questionnaire scores (e.g., HAMD and QIDS-C) rather than biomarkers. There is increasing understanding that neuropsychiatric diseases such as MDD, like many other medical conditions, may be heterogeneous at the molecular level, making accurate prediction of drug response challenging. In an attempt to help address this challenge, an unsupervized learning algorithm using Gaussian Mixture Models (GMMs) was developed to stratify patients based on the similarity of their overall symptom severity at baseline as well as at the 4 and 8 weeks time points in the PGRN-AMPS SSRI trial. Three distinct clusters of patients were identified algorithmically. The number of clusters to be formed was not pre-specified. Instead, the GMM clustering used information-theoretic criteria to determine the minimum number of clusters sufficient to recreate the underlying distribution of depression severity scores at baseline, 4 and 8 weeks. That clustering was replicated using data from the STAR*D antidepressant trial and the clustering behavior at 8 weeks was found to reflect existing definitions of clinical outcomes in MDD–specifically Remission and lack of Remission, or Response and lack of Response. The clusters showed no associations with sociodemographic or clinical factors, as well as no significant differences in plasma drug levels at 4 and 8 weeks. However, the clusters did display sex differences in metabolomic concentrations across all time-points and treatment outcomes—which motivated our development of sex-specific machine learning models to predict SSRI response (Athreya et al., 2017). By doing that, instead of knowing sex as an important predictor of treatment outcomes, it was possible to study the varying degrees of contribution of SNPs to predicting outcomes in men and women separately–setting up a novel way to prioritize SNP selection for future functional studies. However, it should be emphasized that the clusters themselves were not used to predict SSRI response with machine learning approaches due—in part—to their relatively small sample sizes.

**FIGURE 5:**
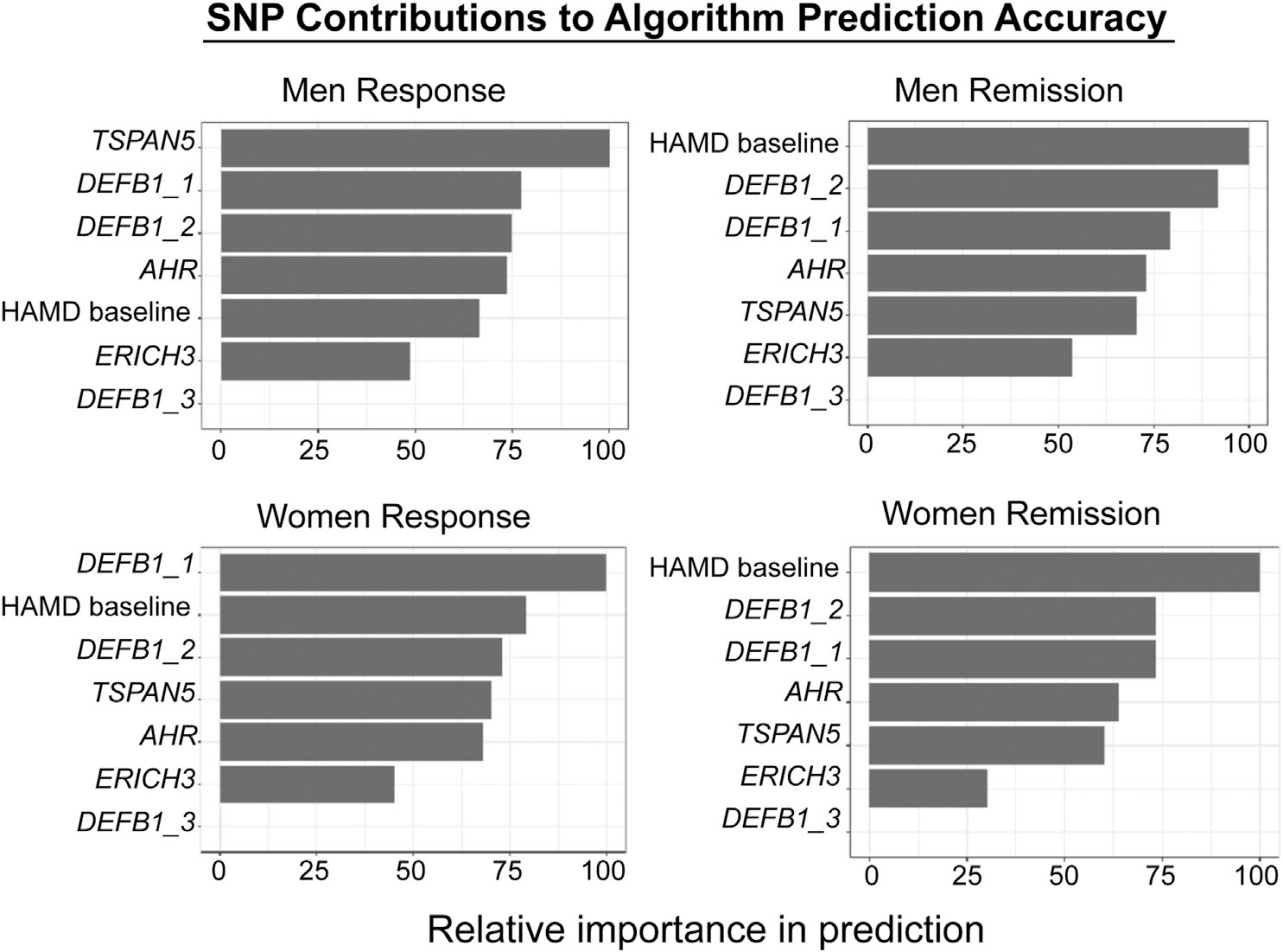
Machine learning algorithm prediction accuracy. The figure shows the relative contributions of individual SNPs and HAMD scores to the predictive accuracy of the algorithm. The X-axis reflects the sensitivity of predictions to variation in the predictor variable—i.e., the higher the variable importance, the larger is the chance of wrong predictions if there is significant variance in the predictor. The figure was adapted from Athreya et al., 2019b. Response and Remission are defined in the text.

The biological measures described in this review, i.e. the SNPs identified during the GWA studies, SNPs associated with metabolites which were themselves associated with clinical response, together with clinical variables such as baseline severity as evaluated by physicians, were incorporated into a supervised machine learning model to predict SSRI response (Athreya et al., 2018; Athreya et al., 2019b). Specifically, random forests were trained to predict sex-specific remission vs. non-remission or response vs. non-response, where the input data were baseline depression severity and genotypes of pharmacogenomic SNPs. The models were trained with repeated cross-validations using the Mayo Clinic PGRN-AMPS data, and were validated using independent samples from the STAR*D (for QIDS-C) and ISPC (for HAMD) studies. The predictive accuracy of the algorithm using clinical data alone was 56%. By including the SNPs in *ERICH3, TSPAN5, DEFB1*, and *AHR*, it was possible to achieve a balanced predictive accuracy of 76%. The prediction accuracy was then validated and replicated using data for MDD patients treated with citalopram/escitalopram in the STAR*D (n = 467) and ISPC (n = 165) trials. Figure 5 shows graphically the contribution of each of the SNPs to the predictive accuracy of the algorithm in men and women separately. The x-axis for Figure 5 reflects the sensitivity of predictions to variation in the predictor variable—i.e., the higher the importance of the variable, the larger is the chance of incorrect predictions if there is significant variance in the predictor. Machine learning approaches can also serve as discovery tools for candidate genes and SNPs, since they make it possible to evaluate the relative contribution of each SNP/gene to prediction accuracy. Those candidate SNPs/genes can then be studied in detail in the laboratory using functional genomic techniques. Finally, by the inclusion of additional candidate genes/SNPs—a process that is already underway for this algorithm—it should be possible to further increase the predictive accuracy of the algorithm. One of the goals of this series of studies, studies that grew out of the application of “pharmaco-omics” to SSRI response in MDD patients, would be the development of a predictive algorithm which—prior to the initiation of SSRI therapy—could be implemented clinically to assist in the selection of therapeutic approaches for patients suffering from MDD (Athreya et al., 2018; Athreya et al., 2019a).

## Conclusion and Future Directions

In this brief overview, we have described the application—over a period of years—of a multi-omics research strategy as one possible approach to address a major challenge in neuropsychiatric research, the relative lack of biological phenotypes and the heterogeneity inherent in complex psychiatric phenotypes. The series of studies described here have focused on SSRIs, first-line pharmacotherapy for MDD, a common psychiatric disorder that affects millions of people worldwide (Alonso et al., 2004; Bromet et al., 2011). The application of multiple omics, particularly utilizing “targeted” metabolomics data to identify metabolites associated with clinical outcomes and then using the identified metabolites to conduct exploratory GWA studies, followed by functional genomics, led to the discovery of novel genes and novel gene function related to variability in SSRI response among patients suffering from MDD. Furthermore, the application of machine learning to develop a predictive algorithm was made possible by merging biological discoveries (i.e. genes and SNPs) with clinical variables to enable prediction of drug response with an accuracy that may, eventually, have clinical utility. Machine learning approaches could also serve as a discovery tool to scan across a series of SNPs/genes to identify the most important contributors to variation in drug response so that those genes and SNPs can be studied in detail in the laboratory. In this way, the drug—SSRIs—will have served as a molecular probe for further investigation into disease pathophysiology by revealing novel genes with unanticipated functions. In future studies, the pharmaco-omics approach described here (see Figure 1) might be applied to other classes of pharmacotherapy in depression, including the use of drugs such as ketamine, or to the study of other complex, heterogeneous neuropsychiatric diseases.

## Author Contributions

TTLN and RW wrote the manuscript. All of the other authors edited the manuscript and provided critical suggestions and modification.

## Funding

This work was supported in part by National Institutes of Health grants R01 GM28157, R01 AA27486, U19 GM61388, K01 AA28050, P20 AA017830, NSF Award #2041339 and by the Mayo Clinic Center for Individualized Medicine. Any opinions, findings, conclusions or recommendations expressed in this material are those of the author(s) and do not necessarily reflect the views of the NSF and NIH.

## Conflict of Interest

RW is a co-founder of and stockholder in OneOme LLC, a pharmacogenomic decision support company.

The remaining authors declare that the research was conducted in the absence of any commercial or financial relationships that could be construed as a potential conflict of interest.
